# A Rare Case of Portal Hypertension and Ascites Following Intrauterine Fetal Death: A Case Report

**DOI:** 10.7759/cureus.55364

**Published:** 2024-03-01

**Authors:** Aizaz A Shah, Vemparala Priyatha, Yamna Ali, Abdul Wahab, Mahejehan A Salarzai, Junaid Ahmad, Noman Salih

**Affiliations:** 1 Internal Medicine, Hayatabad Medical Complex Peshawar, Peshawar, PAK; 2 Internal Medicine, All India Institute of Medical Sciences, Bhubaneswar, Bhubaneswar, IND; 3 Neurology, Rehman Medical Institute, Peshawar, PAK; 4 Medicine, Hayatabad Medical Complex Peshawar, Peshawar, PAK

**Keywords:** therapeutic paracentesis, esophageal varices, dilated portal vein, stillborn, ascites, idiopathic portal hypertension

## Abstract

We present a rare case of a 25-year-old woman who developed idiopathic portal hypertension and ascites four days after delivering a stillborn child at term. She had no previous liver illness or risk factors for portal vein thrombosis. Investigations revealed a dilated portal vein, esophageal varices, and high serum-albumin gradient ascites, all of which point to a presinusoidal etiology of portal hypertension. There was no indication of cirrhosis, hepatic or portal vein thrombosis, metabolic or autoimmune liver diseases, or persistent infections. She was treated with antibiotics, diuretics, and beta-blockers, and she underwent a therapeutic paracentesis. The etiology of her portal hypertension remains undetermined. Idiopathic portal hypertension is a rare condition of unknown etiology, characterized by portal hypertension without cirrhosis or thrombosis. It is linked to several risk factors and histological abnormalities, and it can be accompanied by portal hypertension consequences, such as variceal hemorrhage and ascites. The diagnosis is made using clinical criteria and the elimination of alternative causes of portal hypertension. Management is mostly symptomatic, intending to avoid and treat portal hypertension consequences. The prognosis varies according to the underlying etiology and presence of complications.

## Introduction

Portal hypertension is a disorder marked by elevated pressure in the portal venous system, which can result in problems such as variceal hemorrhage, ascites, and hepatic encephalopathy [1]. Cirrhosis is the most prevalent cause of portal hypertension, closely followed by portal vein thrombosis [1]. Other reasons include congenital portal vein abnormalities, schistosomiasis, and cancer [2]. Portal hypertension is rare in pregnancy and the postpartum period, and it is usually associated with preexisting liver disease, hypercoagulable states, or sepsis [3].

We discuss the case of a young woman who acquired portal hypertension and ascites after giving birth to a stillborn child at full term for no obvious reason. Intrauterine fetal demise (IUFD) occurs when a fetus dies in utero after 20 weeks of gestation [4]. The prevalence of IUFD varies greatly between areas and nations, ranging from 2.5 to 8.8 per 1,000 newborns [5]. The causes of IUFD are multifactorial and include maternal, fetal, and placental factors. Some of the common causes are fetal growth restriction, congenital anomalies, infections, placental abruption, umbilical cord accidents, and maternal medical disorders. IUFD can have a significant psychological and emotional impact on the mother and the family, as well as potential physical complications, such as coagulopathy, infection, and hemorrhage.

The relationship between IUFD and portal hypertension is unclear and poorly understood. There are a few hypotheses describing the underlying mechanisms, but these are speculative and not supported by the available evidence. Some of the proposed hypotheses are a hypercoagulable state induced by pregnancy or fetal death, leading to portal vein thrombosis; a septic complication of fetal death, leading to liver abscess or hepatic vein thrombosis (Budd-Chiari syndrome); a hemodynamic alteration due to fetal death, leading to portal hypertension; a metabolic disorder due to fetal death, such as hemolysis elevated liver enzymes and low platelet count (HELLP) syndrome, acute fatty liver of pregnancy, leading to liver dysfunction and portal hypertension; and a genetic or autoimmune disorder affecting the liver, such as autoimmune hepatitis, Wilson’s disease, or primary biliary cholangitis, leading to portal hypertension [6].

In this case report, we described an uncommon and confusing presentation of portal hypertension and ascites in a young woman who had no history or risk factors for liver disease or portal vein thrombosis. The onset of her symptoms was four days after delivering a stillborn fetus at term, which raises the possibility of a causal relationship between the two events. However, the exact mechanism of how intrauterine fetal death could lead to portal hypertension is unknown. The patient was diagnosed with idiopathic portal hypertension, a rare illness with no known cause that is distinguished by portal hypertension but no cirrhosis or thrombosis. She was treated symptomatically and had a successful outcome, although the cause of her portal hypertension is unknown. Further research is needed to understand the pathogenesis and therapy of this uncommon and confusing illness.

## Case presentation

At 40 weeks of gestation, a 25-year-old female with gravida 4 para 3 presented to the hospital's emergency department with lethargy and reduced fetal movements. She had three prior normal vaginal births with no known medical issues. She had no history of drinking, drugs, or blood transfusions. She has not had any family history of liver disease or coagulopathy. She seemed pallid, tachycardic, and hypotensive. Her abdomen appeared swollen and stiff, with no evidence of peritonitis. Fetal heart sounds were absent. An ultrasound scan confirmed intrauterine fetal death and presented no abnormalities of the placenta or the uterus. She was transferred to the gynecology ward, where she underwent an induced vaginal delivery of a macerated male fetus weighing 3.2 kg. The placenta was intact and normal. She received an oxytocin infusion and intravenous fluids. She had no postpartum hemorrhage or infection. She was observed for three days and was planned for discharge.

On the fourth postnatal day, she complained of abdominal distension and edema in both lower legs. She denied experiencing any stomach discomfort, fever, nausea, vomiting, or changes in bowel habits. She has no history of trauma or surgery. She had no signs of hepatic encephalopathy, such as disorientation, asterixis, or fetor hepaticus. On exam, she was found to be afebrile, having a blood pressure of 150/100 mmHg, a heart rate of 100 beats per minute, with a respiratory rate of 20 breaths per minute. Her oxygen saturation level in indoor air was 96%. Her abdomen was grossly distended, with an everted umbilicus, dilated abdominal wall veins, and a positive fluid thrill. She had pedal pitting edema up to the thighs. Her liver and spleen were not palpable. She had no signs of jaundice, spider nevi, palmar erythema, or caput medusae. Her neurological examination was normal. She was shifted to the medical high-dependency unit for further workup.

The patient's blood tests (Table 1) revealed that most of her readings were normal, except for low albumin. Her liver function tests, renal function tests, electrolytes, iron studies, thyroid profile, autoimmune profile, coagulation profile, fibrinolytic indicators, viral serology, and urine tests (urine routine examination and urinary albumin to creatinine ratio) all exhibited normal results. Her plasma ceruloplasmin levels and 24 hours of urinary copper were also normal, ruling out Wilson’s disease. Both her blood and urine cultures came out negative for infection.

**Table 1:** Baseline investigations Abbreviations: BUN, blood urea nitrogen; Creat, creatinine; ALT, alanine aminotransferase; AST, aspartate aminotransferase; ALP, alkaline phosphatase; Na, sodium; K, potassium; RBS, random blood sugar; LDL, low density lipoprotein; TAG, triacyl glycerol; WBC, white blood cells; Hb, hemoglobin; MCV, mean cell volume

Test	Patient's value	Reference value
ALT (U/L)	43	10-50
AST (U/L)	12	8-33
ALP (U/L)	120	<390
Total bilirubin (mg/dL)	0.5	0.1-1
Troponin I (ng/mL)	12.09	0-0.04
Creat (mg/dL)	0.59	0.3-0.9
BUN (mg/dL)	39	18-45
TAG (mg/dL)	95	<150
LDL cholesterol (mg/dL)	84	<100
K (mmol/L)	3.8	3.5-5.1
RBS (mg/dL)	145	100-125
Na (mmol/L)	144	135-150
WBCs (/µL)	9,000	4,000-11,000
Hb (g/dL)	12.5	11.5-17.5
MCV (fL)	80.8	80-100
Platelets (/µL)	275,500	150,000-450,000
Albumin (g/dL)	2.4	3.5-5.4

Her cardiac testing revealed no abnormalities with her heart rhythm, blood flow, or structure. Her chest X-ray revealed no abnormalities in either her lungs or chest. Her echocardiography revealed normal cardiac function, with no symptoms of elevated blood pressure in the lungs or fluid surrounding the heart. Her abdominal ultrasound revealed a substantial amount of fluid in her abdomen, a slightly enlarged spleen, and a wider portal vein (18 mm), with no blood clots in the portal or hepatic veins. Her fluid testing revealed no infections or tumors. Her ascitic fluid analysis (Table 2) revealed a clear, yellowish fluid with a low protein level, a high difference between albumin levels in the fluid and the blood, a low number of cells, mostly monocytes and lymphocytes (80% monocytes and 20% lymphocytes (normal: < 70% neutrophils)), and a normal sugar level. Her ascitic fluid cytology revealed no suspicious cells.

**Table 2:** Ascitic fluid routine examination Abbreviations: SAAG, serum-ascites albumin gradient

Test	Patient value	Reference value
Proteins (g/dL)	0.8	>2.5
SAAG (g/dL)	1.6	>1.1
Cells (cells/mm^3)	100	<250

**Figure 1:**
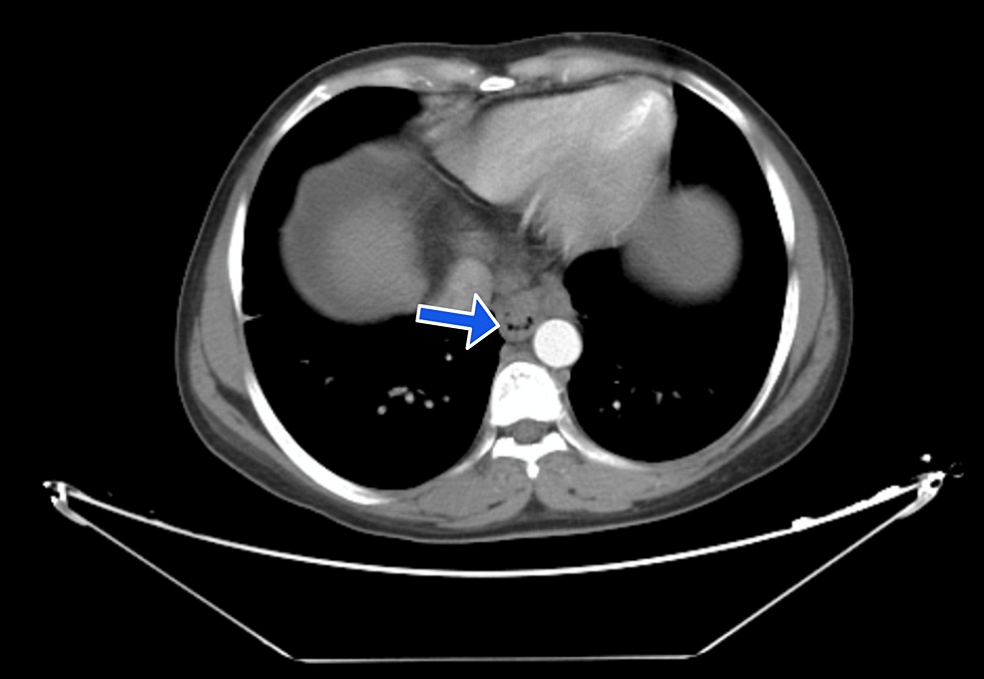
Blue arrow showing esophageal varices

**Figure 2:**
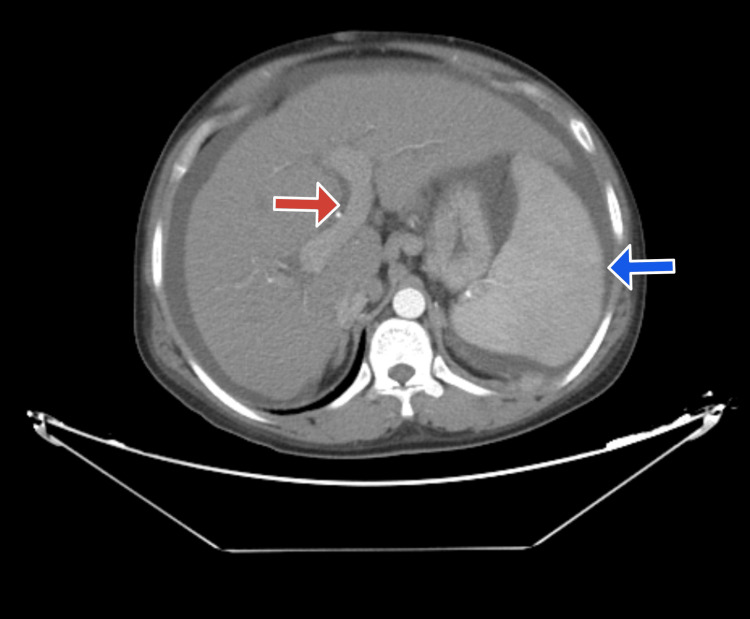
The red arrow points toward a dilated portal vein, while the blue arrow points toward the enlarged spleen

**Figure 3:**
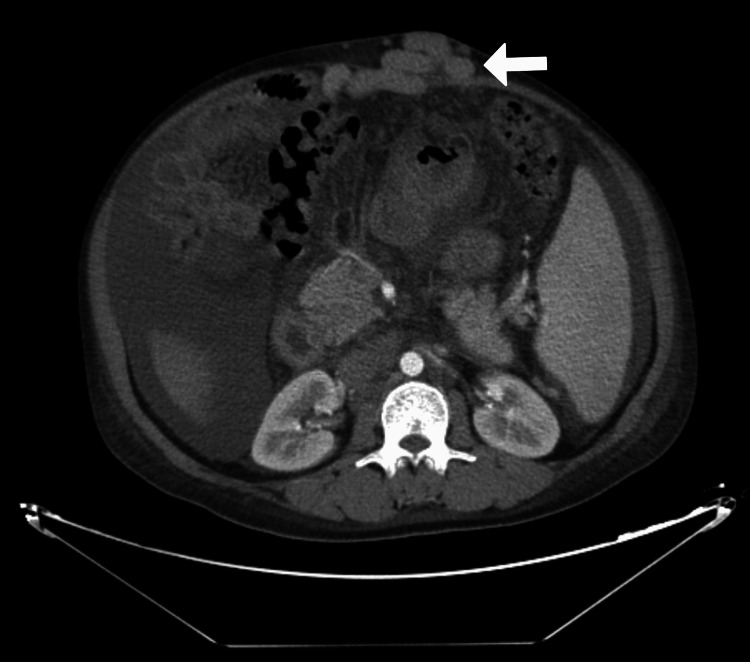
White arrow pointing toward dilated periumbilical veins

Her upper gastrointestinal endoscopy revealed medium-sized esophageal varices, but no bleeding or indications of hemorrhage. Her computed tomography (CT) liver dynamic done specifically for liver pathologies revealed the same results as the ultrasound, with no tumors or abnormalities in the liver, spleen, pancreas, or kidneys, except for the varices (Figure 1); splenomegaly, ascites, and dilated portal vein (Figure 2); and paraumbilical veins (Figure 3).

She was diagnosed with portal hypertension and ascites of unknown etiology. She was treated with oral metronidazole 400 mg thrice a day, as a prophylaxis to prevent any gynecological infection such as endometritis, as she was three days postpartum after her assisted vaginal delivery. Moreover, she was given oral spironolactone 50 mg and furosemide 40 mg once daily for her ascites, intravenous dextrose saline 1 liter twice a day for her hypovolemia, oral proton pump inhibitors (capsule esomeprazole 40 mg once daily on an empty stomach) for her varices, and oral carvedilol 6.25 mg once daily to prevent the occurrence of any bleeding from the gastric varices seen on endoscopy. She underwent a therapeutic paracentesis, which involved the evacuation of six liters of ascitic fluid, and was given an albumin infusion of 50 mL once daily for three days as a plasma expander. Her symptoms and signals gradually improved, and she was released from the hospital after 10 days. She was then recommended to visit the gastroenterology and gynecology clinics regularly as well as to have a repeat endoscopy and liver biopsy to determine the reason for her portal hypertension.

## Discussion

This case report reports a surprising presentation of portal hypertension and ascites in a young woman with no prior history of liver illness or portal vein thrombosis [6]. The onset of her symptoms was four days after delivering a stillborn fetus at term, suggesting a possible causal relationship between the two events. However, the exact mechanism of how intrauterine fetal death could lead to portal hypertension is unknown.

The patient was diagnosed with idiopathic portal hypertension, a rare illness with no known cause that is defined by portal hypertension but neither cirrhosis nor thrombosis. The diagnosis was made based on clinical criteria and the elimination of alternative causes of portal hypertension by several procedures. A liver biopsy could have confirmed the diagnosis and revealed the histological features of the disease, but the patient refused to undergo the procedure [7]. The histology of idiopathic portal hypertension is variable and nonspecific, ranging from minor changes to nodular regenerative hyperplasia. It is unclear whether these changes reflect different stages or subtypes of the disease, or whether idiopathic portal hypertension is a heterogeneous entity with different etiologies [8].

The patient was treated symptomatically and had a favorable outcome. She received antibiotics, diuretics, and beta-blockers and underwent a therapeutic paracentesis [2]. The antibiotics were provided as a prophylaxis to prevent any gynecological infection such as endometritis, as she was three days postpartum after her assisted vaginal delivery. The diuretics were provided to reduce the ascites and portal pressure. The beta-blockers were given to prevent the occurrence of any bleeding from the gastric varices seen on endoscopy [9]. The therapeutic paracentesis was performed to relieve the abdominal distension and improve renal function [10]. The patient steadily improved and was discharged from the hospital after ten days. She was recommended to visit the gastroenterology and gynecology clinics regularly as well as to have a repeat endoscopy and liver biopsy to determine the reason for her portal hypertension.

The likely outcome of idiopathic portal hypertension varies according to the underlying etiology and presence of complications [2]. Patients with idiopathic portal hypertension have a greater survival rate than those with cirrhosis; however, it is less than that of the general population [11]. The major consequences of idiopathic portal hypertension are variceal hemorrhage and ascites, both of which may be avoided and managed using the current cirrhotic portal hypertension guidelines [12]. Other side effects include hepatic encephalopathy, hepatopulmonary syndrome, portopulmonary hypertension, and hepatocellular cancer. Idiopathic portal hypertension patients frequently develop portal vein thrombosis, and anticoagulant treatment should be explored in these circumstances. No medication has been investigated to alter the disease's natural course.

This case report exemplifies a diagnostic problem as the female patient developed portal hypertension and ascites after delivering a stillborn child, with no apparent explanation. The conceivable pathways by which intrauterine fetal mortality may cause portal hypertension are speculative and unsupported by existing research. This individual was diagnosed with idiopathic portal hypertension, a rare illness with no known cause that is defined by portal hypertension but neither cirrhosis nor thrombosis. She was treated symptomatically and had a positive prognosis, although the cause of her portal hypertension is unknown. Further research is needed to understand the pathogenesis and treatment of this rare and confusing illness.

## Conclusions

This case poses a diagnostic challenge, as the patient developed portal hypertension and ascites following IUFD at term, without any identifiable cause. The potential mechanisms of how IUFD could induce portal hypertension are hypothetical and not corroborated by the available evidence. The patient was managed symptomatically and had a favorable outcome, but the etiology of her portal hypertension remains undetermined. Further research is needed to understand the biology and treatment of this rare and complex illness.
